# Protective Effects of Curcumin Ester Prodrug, Curcumin Diethyl Disuccinate against H_2_O_2_-Induced Oxidative Stress in Human Retinal Pigment Epithelial Cells: Potential Therapeutic Avenues for Age-Related Macular Degeneration

**DOI:** 10.3390/ijms20133367

**Published:** 2019-07-09

**Authors:** Chawanphat Muangnoi, Umar Sharif, Pahweenvaj Ratnatilaka Na Bhuket, Pornchai Rojsitthisak, Luminita Paraoan

**Affiliations:** 1 Pharmaceutical Chemistry and Natural Products Program, Faculty of Pharmaceutical Sciences, Chulalongkorn University, Bangkok 10330, Thailand; 2 Natural Products for Ageing and Chronic Diseases Research Unit, Chulalongkorn University, Bangkok 10330, Thailand; 3 Department of Eye and Vision Science, Institute of Ageing and Chronic Disease, University of Liverpool, Liverpool L7 8TX, UK; 4 Department of Food and Pharmaceutical Chemistry, Faculty of Pharmaceutical Sciences, Chulalongkorn University, Bangkok 10330, Thailand

**Keywords:** age related macular degeneration, retinal pigment epithelium, oxidative stress, curcumin, curcumin diethyl disuccinate

## Abstract

Oxidative stress-induced damage to the retinal pigmented epithelium (RPE), a specialised post-mitotic monolayer that maintains retinal homeostasis, contributes to the development of age-related macular degeneration (AMD). Curcumin (Cur), a naturally occurring antioxidant, was previously shown to have the ability to protect RPE cells from oxidative stress. However, poor solubility and bioavailability makes Cur a poor therapeutic agent. As prodrug approaches can mitigate these limitations, we compared the protective properties of the Cur prodrug curcumin diethyl disuccinate (CurDD) against Cur in relation to oxidative stress induced in human ARPE-19 cells. Both CurDD and Cur significantly decreased H_2_O_2_-induced reactive oxygen species (ROS) production and protected RPE cells from oxidative stress-induced death. Both drugs exerted their protective effects through the modulation of p44/42 (ERK) and the involvement of downstream molecules Bax and Bcl-2. Additionally, the expression of antioxidant enzymes HO-1 and NQO1 was also enhanced in cells treated with CurDD and Cur. In all cases, CurDD was more effective than its parent drug against oxidative stress-induced damage to ARPE-19 cells. These findings highlight CurDD as a more potent drug compared to Cur against oxidative stress and indicate that its protective effects are exerted through modulation of key apoptotic and antioxidant molecular pathways.

## 1. Introduction

Age-related macular degeneration (AMD) is the leading cause of irreversible blindness that affects the elderly population in the developed world [1,2]. This disease is characterised by loss of central vision due to the progressive degeneration of the macula. AMD presents itself in two forms: dry form (atrophic, slow progressing) and a more severe wet (neovascular) form. Despite affecting millions of people, the treatments that exist for AMD are still limited and restricted to the wet form. Continued intraocular injection is a common treatment for prevention of wet form progression [3]. On the other hand, no current treatment exists for the dry form of AMD. Thus, the discovery and development of effective treatments are needed to prevent or delay AMD progression.

A primary event that occurs in AMD is represented by pathological changes at the site of a specialised post-mitotic cell layer called the retinal pigment epithelium (RPE) located between the choroidal vasculature and the light sensitive photoreceptors. The RPE performs many important functions that sustain retinal health including visual cycle involvement, digestion of spent photoreceptor outer segments (POS) as well as the establishment of blood-retinal barrier [4]. Due to these vital roles, abnormality of the RPE has adverse effects on the retina and causes vision loss. Evidence implicates oxidative stress in RPE cells, which subsequently leads to impaired function as well as cell death, in AMD pathophysiology [5,6,7,8,9,10].

The macula is exposed to high levels of oxygen and light which makes the RPE in this region particularly prone to the generation of reactive oxygen species (ROS) and oxidative damage [11]. As RPE cells are highly metabolically active, they contain an enriched mitochondria population to help meet their high-energy requirements. The vital process of oxidative phosphorylation that produces the cellular energy source adenosine triphosphate (ATP) leads to generation of high amounts of ROS [12]. The ROS produced not only damage the mitochondria it is generated from, but are also released into the cytoplasm where they act on neighboring mitochondria, causing amplification of ROS levels [13,14,15]. Oxidants such as hydrogen peroxide (H_2_O_2_) also react with iron in lysosomes in a process referred to as the Fenton reaction, which leads to the generation of further ROS. In addition, phagocytosis of POS is also responsible for producing ROS [16].

ROS exert their damaging effects by causing biomolecules such as proteins to misfold, which subsequently causes cellular dysfunction [7]. Removal of toxic misfolded and damaged proteins from the cell is critical for cell survival, particularly in post-mitotic cells such as the RPE [17,18]. ROS also causes the activation of mitogen activated protein kinases (MAPKs) such as extracellular signal-regulated kinase (ERK), p38 mitogen-activated protein kinase (p38), and c-Jun N-terminal kinase (JNK). These MAPKs play an important role in the apoptosis and proliferation of RPE cells via the modulation of apoptosis-associated proteins, namely, Bax (pro-apoptotic) and Bcl-2 (anti-apoptotic) proteins [19,20,21]. Therefore, protection of RPE cells from oxidative damage may be an effective therapeutic strategy against AMD development.

Under normal physiological conditions, the collective effort of endogenous antioxidant defense mechanisms neutralizes cellular damage by ROS. RPE cells contain a pigment called melanin that serves a photoprotective role by absorption of scattered light and scavenging ROS [22,23]. RPE cells also contain a wide range of anti-oxidants such as catalase, glutathione peroxidase, superoxide dismutase, heme oxygenase-1 (HO-1), NADPH dehydrogenase quinone 1 (NQO1) as well as vitamins C and E, which scavenge and decompose ROS [11,24,25]. With age however, the ability of RPE cells to counteract or utilise these ROS diminishes and leads to oxidative stress [26]. For example, a decline in total pure melanin granules is observed in RPE cells with age [27,28]. Decreased activity of antioxidant enzymes is also observed in ageing and in AMD eyes [29].

**Figure 1:**
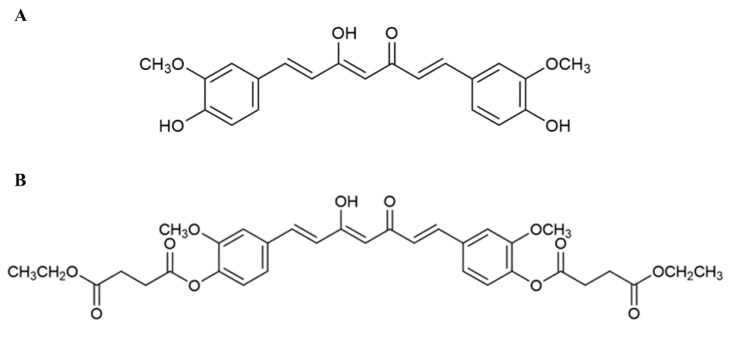
Structure of therapeutic agents used in the present study. (**A**) Curcumin (Cur); (**B**) Curcumin diethyl disuccinate (CurDD).

Several studies have shown protective effects of dietary antioxidants on reducing the risk of AMD [30,31,32]. Curcumin (Cur) (Figure 1A), a major bioactive compound of turmeric (*Curcuma longa* L.) which has antioxidant activity [33,34,35], has been shown to have health benefits for diseases such as cancer, arthritis and Alzheimer’s disease (AD) [36]. Notably, in vitro studies have shown Cur to improve cell viability and decrease apoptosis and oxidative stress in RPE via alterations of apoptosis-associated proteins and antioxidant enzymes [37,38,39]. Cur also inhibits upregulation of inflammatory genes in a light-induced retinal degeneration rat model as well as protecting retinal cells from oxidative induced cell death [40]. Despite its positive effects, one major limitation of the use of Cur as a therapeutic agent is its poor bioavailability [41]. A prodrug approach can be used to enhance pharmacological properties by improving physicochemical and biopharmaceutical properties such as aqueous solubility, stability, and bioavailability [42,43].

In our group, we synthesized a succinate ester prodrug of curcumin called curcumin diethyl disuccinate (CurDD) (Figure 1B), and have shown this to be more stable at pH 7.4. compared to Cur [44]. In addition, CurDD can also be hydrolyzed to active metabolite curcumin by esterase enzymes in plasma [44]. Therefore, CurDD could be a potential therapeutic agent for the prevention of AMD development via its antioxidant activity against oxidative stress-induced RPE injury. In the present study, we evaluated and compared the protective effect of Cur and CurDD on oxidative stress-induced by H_2_O_2_ in RPE cells and explored the underlying molecular mechanisms by which these drugs exert their effect.

## 2. Results

### 2.1. Long-Term Differentiated ARPE-19 Cells Display More Native RPE Characteristics Compared to Undifferentiated ARPE-19 Cells

Due to the limited availability of human donor eyes, primary RPE human cells, a physiologically relevant model to use when studying RPE function, are difficult to obtain. Moreover, these primary cells display donor variability that can lead to difficulty in interpretation of data. Protocols to differentiate human RPE cells from stem cells have recently been developed and provide an important model for RPE study. However, both these models can lose their RPE characteristics after a few passages in culture. Hence, the use of cell lines remains an important source for research studies. In relation to RPE, ARPE-19 cells are the most commonly used model experimental cells, including as a model to study oxidative stress [5,45]. One drawback of using the ARPE-19 cell line is that these cells no longer exhibit many differentiated characteristics such as the cobblestone appearance, polarity and expression of RPE markers as first described 20 years ago [46]. Recently, studies have shown that media conditions and length of culture time allows ARPE-19 cells to obtain a more native, physiological state [47,48]. In the present study, ARPE-19 cells grown in specialised differentiation DMEM media for 3 months were compared to cells grown in standard DMEM/F12 media.

**Figure 2:**
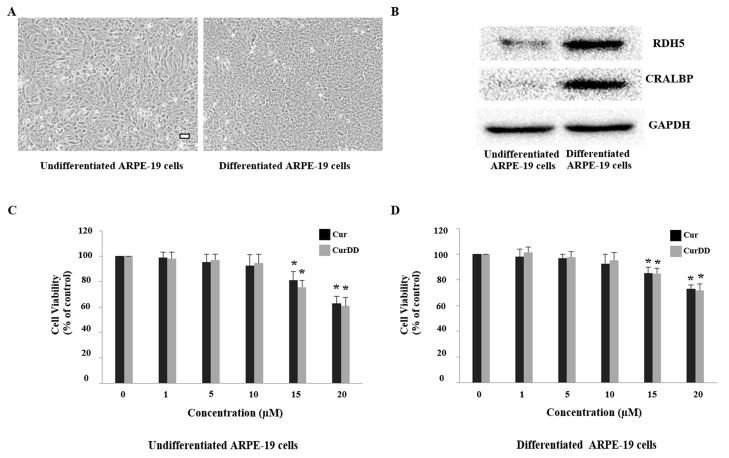
Effect of Cur and CurDD on cell viability of undifferentiated and differentiated ARPE-19 cells. (**A**) Morphology by phase contrast microscopy of undifferentiated ARPE-19 and 3-month differentiated ARPE-19 cells. Scale bar represents 100 µm; (**B**) Protein levels of RPE-specific markers RDH5 and CRALBP were assessed by immunoblotting in undifferentiated and differentiated ARPE-19 cells. GAPDH immunodetection was used as a loading control; (**C**) Undifferentiated and (**D**) differentiated ARPE-19 cells were treated with different concentrations (range 1 to 20 µM) of Cur or CurDD for 24 h after which cell viability was measured using MTT assay. Graphs represent average cell viability (mean ± SD values, *n* = 4; One-Way ANOVA test, * *p* ≤ 0.05 vs control group).

As observed, cells differentiated for 3 months exhibited a more cobblestone appearance and were more tightly packed compared to undifferentiated cells that are longer and more fibroblastic-like in appearance (Figure 2A). Furthermore, the expression of RPE specific markers, retinol dehydrogenase-5 (RDH5) and cellular retinaldehyde binding protein (CRALBP) [46,47] was also examined in the cells by immunoblotting (Figure 2B). Data demonstrated that both undifferentiated and differentiated ARPE-19 cells express RDH5 and CRALBP proteins but higher levels are observed for differentiated cells. The higher RPE marker expression observed in the long-term cultures shows a more differentiated state making them more physiologically relevant. Nevertheless, due to the detection of protein expression of specific RPE markers in undifferentiated and differentiated cells, both models were used for subsequent experiments and further compared.

### 2.2. Evaluation of Cur and CurDD on Cell Viability of Undifferentiated and Differentiated ARPE-19 Cells.

As several studies have shown high concentrations of Cur being toxic to cells [49,50], it was imperative to determine an optimal drug concentration that did not itself affect ARPE-19 cell viability in order to evaluate the protective effects of Cur and CurrDD against oxidative stress. Both undifferentiated and differentiated ARPE-19 cells were treated with a range of concentrations (1–20 µM) for 24 h. Data showed that concentrations up to 10 µM for both compounds did not affect cell viability in undifferentiated and differentiated ARPE-19 cells (Figure 2C,D). We therefore used the highest concentration of Cur and CurDD, 10 µM, for subsequent experiments to ensure effective and sustained drug activity over the period of treatment.

### 2.3. Differentiated ARPE-19 Cells Are More Sensitive to H_2_O_2_-Induced Oxidative Stress than Undifferentiated ARPE-19 Cells

**Figure 3:**
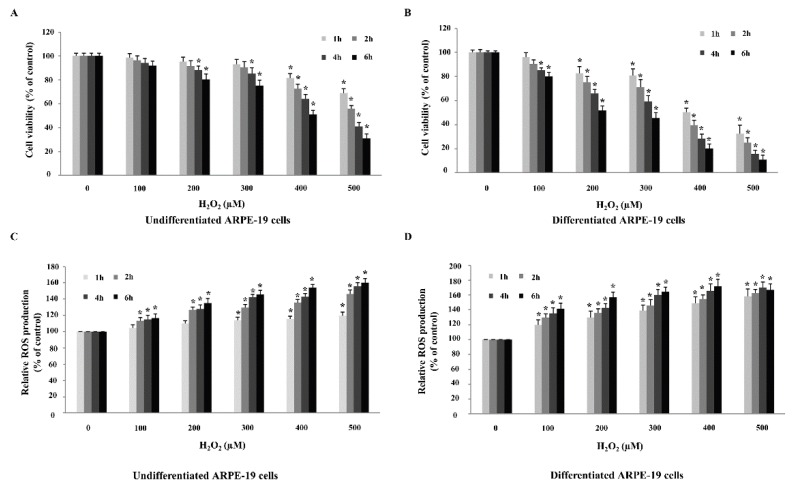
Evaluation of H_2_O_2_ treatment and exposure time needed for reactive oxygen species (ROS) generation and oxidative stress-induced ARPE-19 cell death. (**A**) Undifferentiated and (**B**) differentiated ARPE-19 cells were treated with different concentrations of H_2_O_2_ (within the range 100–500 µM) over a time course of 0–6 h after which cell viability was measured using MTT assay. ROS production was also measured under the same experimental conditions for both (**C**) undifferentiated and (**D**) differentiated ARPE-19 cells. Graphs represent average cell viability and relative ROS production (mean ± SD values, *n* = 4; One-Way ANOVA test, * *p* ≤ 0.05 vs. control group).

We next sought to determine the highest concentration of H_2_O_2_ required to cause an approximately 50% reduction in ARPE-19 cell viability. Undifferentiated and differentiated ARPE-19 cells were treated with a range of H_2_O_2_ concentrations between 100–500 µM over a 1–6 h. Results demonstrated that H_2_O_2_ treatment decreased cell viability and increased ROS production of undifferentiated and differentiated ARPE-19 cells in a dose and time-dependent manner (Figure 3). Interestingly, undifferentiated and differentiated ARPE-19 cells differed in sensitivity to H_2_O_2_ concentration. For undifferentiated cells, H_2_O_2_ treatment for 6 h at a concentration of 400 µM was sufficient to achieve a 50% reduction in cell viability (Figure 3A). On the other hand, differentiated cells were more sensitive to H_2_O_2_ treatment as a lower concentration of 200 µM at the same time point of 6 h caused 50% cell viability reduction (Figure 3B). We therefore used the above determined concentrations and time points for H_2_O_2_ treatment in subsequent experiments for the two cell models.

### 2.4. Cur and CurrDD Exert A Protective Effect against H_2_O_2_-Induced Oxidative Stress in ARPE-19 Cells

**Figure 4:**
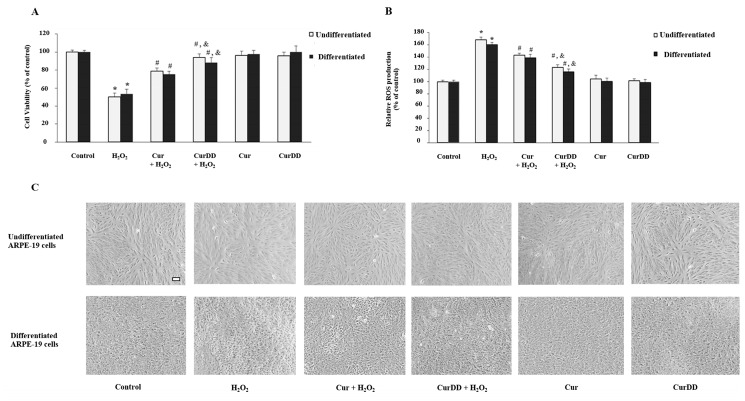
Protective effects of Cur and CurDD against H_2_O_2_-induced ROS production and cytotoxicity in ARPE-19 cells. (**A**) ARPE-19 cells were pre-treated with 10 µM of Cur or CurDD for 24 h, followed by H_2_O_2_ treatment at appropriate concentrations (400 and 200 µM for undifferentiated and differentiate cells, respectively) for 6 h. Cell viability was measured using MTT assay; (**B**) ROS generation was determined by DCFH-DA assay. Graphs represent average cell viability (mean ± SD values, *n* = 4; One-Way ANOVA test, * *p* ≤ 0.05 vs. control group, # *p* ≤ 0.05 vs. H_2_O_2_ group, and & *p* ≤ 0.05 vs. Cur + H_2_O_2_ group); (**C**) Morphology by phase contrast microscopy of undifferentiated ARPE-19 and differentiated ARPE-19 cells under all experimental conditions. Scale bar represents 100 µm.

We next determined and compared the protective effects of CurDD against Cur on oxidative stress in human ARPE-19 cells. Undifferentiated and differentiated ARPE-19 cells were treated with chosen optimal concentrations of H_2_O_2_ for 6 h. In line with previous data, cell viability was reduced (Figure 4A). Simultaneously, ROS production was induced in both cell models (Figure 4B). Pre-treatment of cells with both Cur and CurDD for 24 h significantly protected cells from oxidative stress-induced cell death and ROS production in both models (Figure 4A,B). CurDD showed an increase in the protective effect on cell viability and ROS production in both cell models at a slightly higher extent compared to that of Cur. Drug-treated cells maintained normal morphology, thus supporting the protective effect of Cur and CurDD against H_2_O_2_-induced oxidative damage in the undifferentiated and differentiated ARPE-19 cells (Figure 4C). These findings highlighted CurDD as a more effective protective agent against oxidative stress than Cur.

### 2.5. Cur and CurDD Exert Their Protective Effect against H_2_O_2_-Induced Oxidative Stress via Modulation of the p44/42 MAPK Pathway

**Figure 5:**
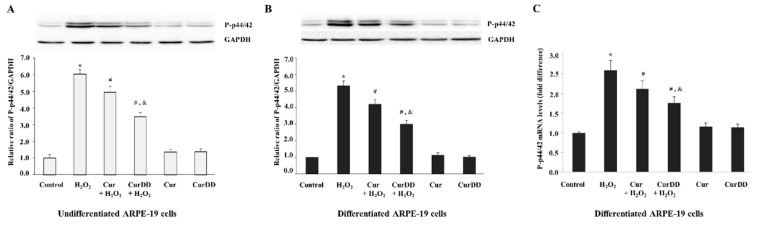
Protective effects of Cur and CurDD against oxidative stress occur through modulation of apoptotic MAPK p44/42 signalling pathway. ARPE-19 cells were pre-treated with 10 µM of Cur or CurDD for 24 h, followed by H_2_O_2_ treatment at appropriate concentrations (400 and 200 µM for undifferentiated and differentiate cells, respectively) for 6 h. Protein levels of phosphorylated P-p44/42 were assessed by immunoblotting in (**A**) undifferentiated and (**B**) differentiated ARPE-19 cells. GAPDH immunodetection was used as a loading control. Representative Western blots shown, with graphs presenting average normalised protein expression; (**C**) mRNA levels of p44/42 were analysed by qPCR. Graph represents average expression normalised against four housekeeping genes as described in Methods. (For both protein and mRNA, data is presented as mean ± SD values, *n* = 4; One-Way ANOVA test, * *p* ≤ 0.05 vs. control group, **^#^** *p* ≤ 0.05 vs. H_2_O_2_ group, and ^&^ *p* ≤ 0.05 vs. Cur + H_2_O_2_ group).

The next part of the study aimed to delineate and understand the molecular mechanisms underpinning the protective effects of Cur and CurDD against oxidative stress in ARPE-19 cells. Previous studies have confirmed that the activation of MAPKs such as p44/42 (ERK1/2) is strongly associated with promotion of H_2_O_2_-induced cell apoptosis [51]. We therefore investigated if the same pathway modulated the H_2_O_2_-induced cell death observed in this present study. Analysis of protein expression, via immunoblotting, showed that after H_2_O_2_ treatment a significant increase in phosphorylated p44/42 was observed in both undifferentiated (Figure 5A) and differentiated (Figure 5B) ARPE-19 cells in comparison with the three control groups (untreated cells, Cur only, and CurDD only cells). This finding suggests that H_2_O_2-_induced cell death observed occurs through ERK1/2 pathway. Interestingly, in cells which were pretreated with Cur and CurDD, the increase in phosphorylated p44/42 was reduced in H_2_O_2_-treated cells compared to what was observed in H_2_O_2_-treated cells without drug pretreatment for both undifferentiated and differentiated ARPE-19 cells. In order to see if the p44/42 changes were due to either increase in transcriptional levels or to increase in phosphorylation, mRNA expression was determined by real time qPCR. mRNA expression for all conditions followed the same pattern as observed for the P-p44/42 protein levels (mRNA expression data shown for differentiated ARPE-19 cells) (Figure 5C), suggesting that the P-p44/42 changes were due to changes in the total amounts of the protein pool rather than post-translational changes. Furthermore, the results also highlighted that Cur and CurDD exert their protective effects through modulation of the ERK1/2 pathway. When comparing Cur to CurDD, pretreatment with CurDD caused an even more significant decrease in expression of P-p44/42 than Cur in the oxidative stress-induced cell models. This again emphasizes that CurDD is a more effective and efficient drug compared to Cur in relation to its ability to reverse oxidative stress-induced response.

### 2.6. Cur and CurDD Inhibit Apoptosis by Modulation of Bax and Bcl2 Expression in Oxidative Stressed ARPE-19 Cells

**Figure 6:**
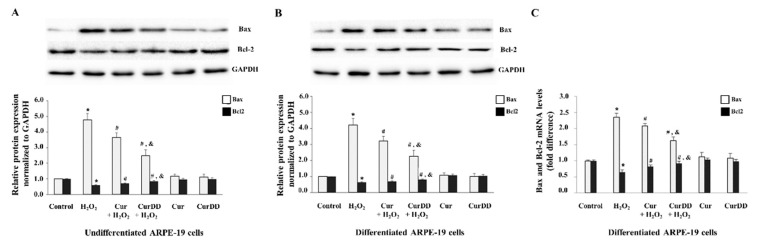
Protective effects of Cur and CurDD against oxidative stress occurs through modulation of apoptotic regulatory molecules Bax and Bcl-2. ARPE-19 cells were pre-treated with 10 µM of Cur or CurDD for 24 h, followed by H_2_O_2_ treatment at appropriate concentrations (400 and 200 µM for undifferentiated and differentiate cells, respectively) for 6 h. Protein levels of Bax and Bcl2 were assessed by immunoblotting in (**A**) undifferentiated and (**B**) differentiated ARPE-19 cells. GAPDH immunodetection was used as a loading control. Representative Western blots shown, with graphs presenting average normalised protein expression; (**C**) mRNA levels of Bax and Bcl2 were analysed by qPCR. Graph represents average expression normalised against four housekeeping genes as described in Methods. (For both protein and mRNA, data is presented as mean ± SD values, *n* = 4; One-Way ANOVA test, * *p* ≤ 0.05 vs. control group, **^#^** *p* ≤ 0.05 vs. H_2_O_2_ group, and ^&^ *p* ≤ 0.05 vs. Cur + H_2_O_2_ group).

To gain further understanding of the molecular mechanisms that Cur and CurDD use to provide a protective effect against oxidative stress, we also examined the expression levels of molecules downstream of the ERK1/2 signaling pathway, which are the pro-apoptotic Bax and anti-apoptotic Bcl2 proteins [52,53]. Expression of Bax/Bcl2 was demonstrated by immunoblotting in both undifferentiated and differentiated ARPE-19 cell cultures (Figure 6A,B). Analysis of protein expression showed that the respective H_2_O_2_ treatment significantly and simultaneously increased Bax and decreased Bcl2 levels in both undifferentiated and differentiated cultures compared to the three control groups (untreated cells, Cur only, and CurDD only cells). For both undifferentiated and differentiated cells, pretreatment with Cur and CurDD led to a significant decrease in Bax and significant increase in Bcl2 protein levels in cells that were treated with H_2_O_2_ when compared to only H_2_O_2_ treated cells. The protein expression pattern for both Bax and Bcl2 was also similar to that observed for mRNA expression under the experimental conditions tested (mRNA expression data shown for differentiated ARPE-19 cells) (Figure 6C). Therefore, Cur and CurDD exert their protective effect against oxidative stress-induced cell death via modulation of the Bax and Bcl2 most likely at a transcriptional level. CurDD again was a more effective agent in its protective effect against oxidative stress as shown by its enhanced ability to reverse the oxidative induced alterations of Bax and Bcl2 protein expression when compared to Cur treatment.

### 2.7. Cur and CurDD Exert Their Protective Effect against Oxidative Stress-Induced Cell Death via Modulation of Key Anti-Oxidant Enzymes in ARPE-19 Cells

Two key antioxidant enzymes known to protect against oxidative damage by counteracting high levels of ROS in cells are heme oxygenase (HO-1) and NAD(P)H:quinone oxidoreductase 1 (NQO1) [54,55]. Administration of Cur is known to upregulate protein expression levels of both HO-1 and NQO1 in a mouse model with traumatic brain injury and ameliorate secondary issues associated with injury such as oxidative stress [56]. We therefore examined whether Cur and CurDD modulate HO-1 and NQO1 expression to provide protection to ARPE-19 cells against oxidative stress.

**Figure 7:**
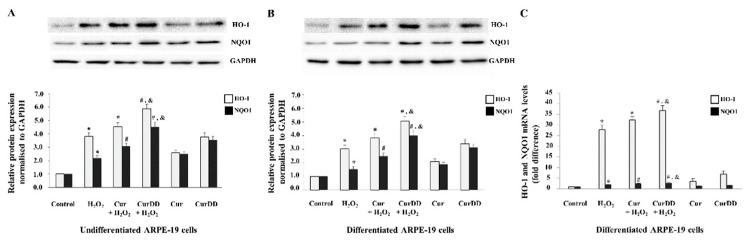
Protective effects of Cur and CurDD against oxidative stress occurs through modulation of antioxidant enzymes HO-1 and NQO1. ARPE-19 cells were pre-treated with 10 µM of Cur or CurDD for 24 h, followed by H_2_O_2_ treatment at appropriate concentrations (400 and 200 µM for undifferentiated and differentiate cells, respectively) for 6 h. Protein levels of HO-1 and NQO1 were assessed by immunoblotting in (**A**) undifferentiated and (**B**) differentiated ARPE-19 cells. GAPDH immunodetection was used as a loading control. Representative Western blots shown, with graphs presenting average normalised protein expression; (**C**) mRNA levels of HO-1 and NQO1 were analysed by qPCR in differentiated ARPE-19 cells. Graph represents average expression normalised against four housekeeping genes as described in Methods. (For both protein and mRNA, data is presented as mean ± SD values, *n* = 4; One-Way ANOVA test,* *p* ≤ 0.05 vs. control group,* *p* ≤ 0.05 vs. control group, **^#^** *p* ≤ 0.05 vs. H_2_O_2_ group, and ^&^ *p* ≤ 0.05 vs. Cur + H_2_O_2_ group).

Analysis of protein expression showed that the H_2_O_2_ treatment significantly increased protein levels of both antioxidant enzymes in both undifferentiated and differentiated cultures compared to the control group without any treatment (Figure 7A,B). Furthermore, pretreatment with Cur and CurDD significantly enhanced protein levels of these two enzymes in the presence of oxidative stress. Interestingly, we observed that Cur or CurDD treatment was itself enough to significantly increase protein expression of HO-1 and NQO1 for both undifferentiated and differentiated control cells. This suggests that the antioxidant system of the RPE can be “primed” by Cur and CurDD pretreatment in order to be equipped against potential oxidative stress inducers. The protein expression pattern observed for all conditions was mirrored by a similar trend of mRNA level (mRNA expression data shown for differentiated ARPE-19 cells) (Figure 7C). In all cases, CurDD was able to provide a more effective response in antioxidant enzyme expression levels compared to Cur.

## 3. Discussion

In the present study we demonstrated for the first time to our knowledge the protective effects of CurDD, a prodrug ester form of Cur, against oxidative stress in human RPE cells. At a molecular level, we showed that both Cur and CurrD exert their protective effects through the modulation of the key apoptotic signaling pathway, p44/42 (ERK), and its downstream effector molecules Bax and Bcl2. In addition, Cur and CurDD were also able to provide protection against oxidative damage by increasing the expression of key antioxidant enzymes HO-1 and NQO1 in ARPE-19 cells. Furthermore, we showed that CurDD provides a better protective effect than Cur against oxidative insult in ARPE-19 cells, thus highlighting the potential use of CurDD as alternative therapeutic agent for AMD, a disease caused by oxidative-induced RPE dysfunction and cell death.

The 3-month differentiated ARPE-19 cellular model used in the present study was based upon recent findings that showed the possibility of obtaining a more differentiated physiologically native RPE state [47,48]. Here, we showed that our 3-month ARPE-19 cell culture, not only exhibited a more cobblestone appearance, but also had higher protein expression levels of RPE specific markers, RDH5, and CRALBP, compared to undifferentiated ARPE-19 cells that were cultured under routine conditions. This highlighted that these cells were more physiologically relevant compared to cells in short-term culture. The differentiated cells used in the present study did however not reach the pigmentation observed in other studies; this may be due different factors such as passage number, surface substrate they are grown on and media used [47,48]. Before comparing the antioxidant effect of Cur and its prodrug CurDD, we noticed that differentiated ARPE-19 cells were more sensitive to oxidative stress, as shown by a lower concentration of H_2_O_2_ treatment needed to kill 50% of cells, compared to undifferentiated cells. A recent study comparing ARPE-19 cells with human stem cell-derived RPE cells, which display native phenotypical characteristics such as pigmentation, polarization, and expression of RPE signature genes, showed that stem cell-derived RPE cells had increased sensitivity to oxidative stress [57]. This collaborates with the data presented here as a more “differentiated” cell model has increased sensitivity to oxidative stress.

Next, we compared the protective effects of Cur and CurDD against oxidative stress in ARPE-19 cells. Cur has been shown to be protective against H_2_O_2_-induced oxidative stress in different cell models such as in bone marrow mesenchymal stem cells, osteoblasts, and cardiomyocytes [58,59], as well as RPE [37]. Therefore, it was hypothesized that the prodrug of Cur, CurDD, would exert a similar effect against oxidative stress. Several publications have reported the presence of intracellular esterase enzymes in ARPE-19 cells [60,61]. Those esterase enzymes including carboxylesterase, acetylcholinesterase, and butyrylcholinesterase can hydrolyze the ester bond of ester prodrugs [60,61]. Cholkar et al. reported the bioconversion of an ester prodrug of ganciclovir to the active metabolite ganciclovir in APRE-19 cells to exhibit an antiviral effect [62]. Additionally, we previously reported that CurDD could be hydrolyzed by carboxylesterase and butyrylcholinesterase in plasma [63]. Therefore, CurDD is suggested to be metabolized to Cur by esterases in APRE-19 cells prior to exerting antioxidant activities. As expected, we observed that undifferentiated and differentiated ARPE-19 cells pretreated with Cur and CurDD alleviated H_2_O_2_-induced ROS production as well as cell death. CurDD exerted a slightly higher potency on the protective effect against oxidative stress compared to Cur-treated cells, as shown by a 10% to 15% increase and decrease in the cell viability and in ROS production, respectively. The antioxidant activities of CurDD at a slightly higher extent compared to those of Cur, in conjunction with its better chemical stability as reported previously [44], suggest that CurDD is an effective prodrug of Cur.

At molecular level, we demonstrated that Cur and CurDD were able to exert their protective effect against oxidative stress through the modulation of the extracellular signal-regulated kinases (ERK) 1/2 (p44/42) signaling pathway. This signaling pathway is involved in functions such as cell proliferation and apoptosis. In general, transient (less than 15 min) activation of this pathway is significant for cell proliferation and survival [64], whereas sustained activation of the p44/42 kinases is able to induce cell death [65]. Activation of p44/42 is able to influence expression of downstream apoptotic regulators Bax (pro-apoptotic) and Bcl-2 (anti-apoptotic) [53]. The balance between Bax and Bcl-2 determines whether apoptosis is triggered [66]. The activation of p44/42 was shown to be strongly associated with promotion of H_2_O_2_-induced cell apoptosis in renal epithelial cells [51]. In addition, Cur treatment has been shown to inhibit apoptosis through modulation of p44/42 signaling [67]. Furthermore, a recent study showed that Cur was able to inhibit apoptosis through modulating Bax/Bcl2 expression and reduced oxidative stress in testes of diabetic rats [68]. Thus, investigating the activation of p44/42 as well as expression of downstream molecules Bax and Bcl-2 informed the understanding of how H_2_O_2_ induces apoptosis in RPE cells. In the present study, H_2_O_2_ treatment led to increased protein levels of the phosphorylation of p-44/42 (P-p44/42) in both undifferentiated and differentiated ARPE-19 cells. This was also accompanied by an increase in protein expression of Bax as well as reduction in protein expression of Bcl-2. These findings showed that cell death observed in RPE cells upon H_2_O_2_ treatment was through the apoptotic pathway. Remarkably, pretreatment with Cur and CurrDD prevented these changes leading to increased cell viability against oxidative stress, with CurDD being more effective in modulating these apoptotic molecules compared to Cur in both RPE cell models.

In addition to apoptotic signaling pathways, we demonstrated that Cur and CurDD were able to influence the expression of antioxidant enzymes HO-1 and NQO1. Specifically, we showed that cells pretreated with Cur and CurDD led to increased expression of HO-1 and NQO1 and thus protected cells from oxidative stress induced by H_2_O_2_ treatment. Moreover, CurDD was able to exert a more potent effect in modulating these antioxidant enzymes when compared to Cur. The influence of these drugs on the expression of antioxidant enzymes was expected as Cur, which is a potent scavenger of ROS [69], can function indirectly as an antioxidant by increasing the expression of these key antioxidant enzymes [70]. In ARPE-19 cells, it had been reported previously that Cur protected ARPE-19 cells against oxidative stress by increasing HO-1 expression and subsequent reduction in ROS levels [37]. Another study showed that administration of Cur upregulated protein expression levels of both HO-1 and NQO1 in a mouse model with traumatic brain injury and ameliorated secondary issues associated with injury such oxidative stress [56]. In the present study, we compared effects of Cur and CurDD on HO-1 and NQO1 expression in ARPE-19 cells under induced oxidative stress condition. It is likely that Cur/CurDD protected ARPE-19 cells from oxidative stress by increasing expression of HO-1 and NQO1, which subsequently reduced ROS levels within the cells.

An interesting observation in the present study was that cells pretreated with Cur or CurDD showed increased protein expression of HO-1 and NQO1 similar to that associated with the initial response in oxidative stressed cells. Furthermore, pretreated cells exposed to H_2_O_2_, showed further increase in levels of these enzymes. The increase of antioxidant enzymes after oxidative stress induction was in line with a study where protein levels of HO-1 increased in ARPE-19 cells after H_2_O_2_ treatment [37]. However, a more recent study showed that H_2_O_2_ treatment significantly reduced the protein expression of enzymes such as HO-1 and NQO1 in ARPE-19 cells [71]. The difference between the studies may be due to the use of different concentrations of H_2_O_2_ as well length of treatment, all of which can influence cellular response. In our study, it may be that initially, without Cur and CurDD pretreatment, the observed increase in the two enzymes upon H_2_O_2_ treatment may be an attempt to counteract the ROS levels within the cells. However, this increase was not enough to overcome the level of oxidative stress within cells shown by the high levels of ROS and cell death observed. Cur and CurDD pretreatment however further enhanced the level of these enzymes to a point where they were able to overcome oxidative insult and thus enabling cells to survive.

The signaling mechanisms that regulate HO-1 and NQO1 expression were not investigated in this study. It has been reported that antioxidants protected oxidative stressed RPE cells through activation of the Akt/Nrf2 signaling pathway, involving the translocation of Nrf2 into the nucleus which results in the expression of several antioxidants such as HO-1 and NQO1 [72,73]. Other studies have shown that Cur exerts its protective effects against oxidative stress through activation of the Nrf2 signaling [74,75]. It is likely that Cur as well as CurDD are able to influence the levels of the antioxidant enzymes through this pathway. This is supported by increased mRNA levels of HO-1 and NQO1 observed in the present study that most likely caused changes in the respective proteins levels.

In summary, the present study evidences that CurDD is a more potent protective antioxidant than Cur against oxidative damage to ARPE-19 cells through suppression of ROS levels and protection against cell death. The molecular mechanisms by which CurDD showed its effects involves modulation of the apoptotic signaling pathway p44/42 and downstream proteins such as Bax and Bcl2 as well as enhancement of antioxidant enzyme levels. The results provide first experimental evidence for CurDD as a promising therapeutic agent for the treatment of AMD.

## 4. Materials and Methods

### 4.1. Chemicals and Reagents

Cur (purity >98% by HPLC) and CurDD (purity >98% by HPLC) were synthesized as previously described [44] and characterized by Nuclear Magnetic Resonance (NMR). Fetal bovine serum (FBS) was obtained from Life Science Production (LSP, Bedford, UK). Dulbecco’s modified Eagle’s Medium/Nutrient Mixture F-12 Ham (DMEM/F-12), Dulbecco’s modified Eagle’s medium (DMEM)- high glucose, L-glutamine sodium pyruvate solution, hydrogen peroxide, 3-[4,5-dimethyltiazol-2-yl]-2,5-diphenyl-tetrazolium bromide (MTT), 2’,7´-dichlorofluorescence diacetate (DCFH-DA), and dimethyl sulfoxide (DMSO) were purchased from Sigma-Aldrich (Sigma, Dorset, UK). All antibodies for western blot were purchased from Cell Signaling Technology (CST, Danvers, MA, USA).

### 4.2. Cell Culture of ARPE-19 cells

An authenticated cell line ARPE-19 (ATCC, Rockville, MD, USA) was used as an experimental model to represent human RPE cells. These cells were routinely maintained and cultured in 1:1 mixture of DMEM/F-12 supplemented with 10% heat inactivated FBS. In order to differentiate ARPE-19 cells to a more native and physiologically relevant state, once confluent, media was switched to a specialised DMEM media which contained high glucose (4.5g/L) supplemented with 1% heat inactivated FBS, 1 mM sodium pyruvate and 2 mM L-glutamine for 3 months [47]. In all cases, media exchange was performed 3 times a week. All experiments were carried out in standard 6-well or 24-well plates, unless stated otherwise. For 6-well plate set-up, undifferentiated and differentiated ARPE-19 cells were seeded at a cell density of 0.3 × 10^6^ and 1.0 × 10^6^ cells/well, respectively. For a 24-well plate set-up, undifferentiated and differentiated ARPE-19 cells were seeded at a cell density of 0.5 × 10^5^ and 1.5 × 10^5^ cells/well, respectively. Cells were then grown for 1 week to obtain a confluent monolayer. These cells were then used for subsequent experiments.

### 4.3. Cell viability (MTT) Assay

Cell viability was measured using the 3-(4, 5-dimethlthiazol-2-yl)-2, 5-diphenyltetrazolium bromide tetrazolium (MTT) assay. The MTT substrate, when incubated with cells, is converted into a purple colored formazan product that is proportional to the amount of viable cells. Therefore, this method is a useful measure of cell viability. After cells had reached their respective experimental time points, media was removed and cells were incubated with phosphate buffer saline (PBS) containing 0.5 mg/ml MTT reagent at 37 °C for 4 h. After incubation, MTT solution was removed from the cells and DMSO was added to solubilize formazan produced from MTT by viable cells. Cells were incubated at 37 °C for 10 min after which absorbance was measured at 540 nm using the SPECTROstarNano microplate reader (BMG LABTECH, Aylesbury, UK).

### 4.4. Evaluation of Cytotoxicity of Cur or CurDD

Undifferentiated and differentiated ARPE-19 cells were cultured in a 24-well plate set up for 1 week. After this, media was removed and cells were washed with excess PBS. After washing, cells were incubated with different concentrations of Cur or CurDD (1, 5, 10, 15, and 20 µM) for 24 h. A DMSO vehicle control well was included in each experiment. After the respective experimental time, cell viability was determined by MTT assay.

### 4.5. Evaluation of Suitable H_2_O_2_ Concentration for Cytotoxicity Induction

Media was removed and cells cultured in a 24-well plate set up as above were washed with excess PBS. After washing, cells were incubated with different H_2_O_2_ concentrations (100, 200, 300, 400, and 500 µM ) in serum free medium at 37 °C for 1, 2, 4, and 6 h. Control cells were cultured in serum free medium without H_2_O_2_. After the respective experimental treatment, cells were washed twice with excess PBS after which cell viability was measured by MTT assay.

### 4.6. Evaluation of the Protective Effect of Cur and CurDD on ARPE-19 Induced Oxidative Stress

Both 6-well and 24-well plate set up was used for this part of the study. After 1 week following differentiation in culture, cells were washed with PBS after which appropriate wells were incubated for 24 h with either Cur or CurDD (both at a concentration of 10 µM) in serum free media. A DMSO vehicle control well was included in the experiment. After pretreatment with Cur or CurDD, both undifferentiated and differentiated cells were washed with PBS and then incubated with appropriate H_2_O_2_ concentrations in serum free medium at 37 °C for 6 h. The 24-well plate set up was used for cell viability measurement using the MTT assay. The 6-well plate set up was used to obtain cell protein lysates for the Western blot analysis, described below.

### 4.7. Evaluation of Reactive Oxygen Species (ROS) Production

DCFH-DA was used to detect and quantify intracellular ROS activity. Undifferentiated and differentiated ARPE-19 cells were seeded into wells of black 96-well, clear bottom plates (Corning, NY, USA) at a cell density of 1.0 and 3.0 × 10^4^ cells/well, respectively, and cultured for 24 h. Media was then removed and cells were washed with excess PBS. After washing, appropriate wells were incubated for 24 h with either Cur or CurDD (both at a concentration of 10 µM) in serum free media. A DMSO vehicle control well was included in each experiment. After the respective experimental time point, media was removed and cells were washed with excess PBS. Next, cells were incubated with 10 µM DCFH-DA in serum free media at 37 °C for 20 min. The cells were then washed with PBS followed by incubation with appropriate concentrations of H_2_O_2_. Finally, cells were washed twice with PBS and ROS production was measured in the plates using the Fluostar Optima plate reader (BMG Labtech, Aylesbury, UK) with the excitation/emission settings of 485 nm/530 nm respectively.

### 4.8. Western Blot Analysis

Undifferentiated and differentiated ARPE-19 cells from a 6-well plate set up were used for preparation of cellular protein lysates. Cell protein lysates were prepared in lysis buffer [76] and subjected to immunoblotting. Once collected, cell lysates were centrifuged at 14,000 g at 4 °C for 10 min. The supernatants were retained and the protein concentrations were determined using the QubitTM protein assay kits (Invitrogen Ltd., Paisley, UK). Equal amounts (40 µg) of protein samples were separated by 10% SDS-PAGE; the resolved proteins were then transferred to Amersham™ Protran^®^ nitrocellulose membrane (Sigma Aldrich, Dorset, UK), after which the membrane was blocked with 5% dry milk. The primary and secondary antibodies used are listed in Table 1. After primary and secondary antibody steps, protein detection was achieved using an enhanced chemiluminescent detection kit (Thermo Scientific, Rockford, IL, USA) followed by imaging on the BioRad ChemiDocTM (BioRad, Hampstead, UK). The results were analyzed by Image Lab software (BioRad, Hampstead, UK) to obtain the corresponding optical density. Band densitometry values obtained were normalised to the loading control (glyceraldehyde 3-phosphate dehydrogenase (GAPDH)).

### 4.9. Real-Time Quantitative PCR (qPCR)

RNA isolation was performed using the RNeasy Plus Mini-Kit (Qiagen, Hilden, Germany). Complementary DNA was synthesized from RNA using the First Strand cDNA Synthesis Kit (Thermo Scientific, Waltham, MA, USA). Quantitative PCR was performed with the MESA BLUE qPCR Mastermix Plus Kit for SYBR assay (Eurogentec, Liege, Belgium). The reactions were run on a Stratagene MX3005P qPCR System (Stratagene, San Diego, CA, USA), with a minimum of three biological replicates for each experimental condition and three technical replicates for each cDNA sample. Primer sets used are listed in Table 2. Final values were expressed relative to a calibrator sample assigned an arbitrary value of 1 and normalized to the expression of four housekeeping genes, beta tubulin, beta-actin (ACTB), GAPDH and ribosomal protein L5 (RPL5) using the efficiency corrected ddCt method. The specificity of amplification reactions was confirmed by melt curve analysis.

### 4.10. Statistical Analysis

All data are presented as the mean values ± standard deviations (SD) of a minimum of three independent experiments. Data analysis was performed using the commercially available software SPSS (Version 16.0). A *p*-value of ≤0.05 was considered statistically significant.

## Figures and Tables

**Table 1:** Antibodies used for the analysis of protein expression.

Antibodies	Dilution
Anti-RDH5 (Abcam) Anti-CRALBP (Abcam) Anti-Phospho-p44/42 (Cell Signalling, Hertfordshire, UK) Anti-Bax (Cell Signalling, Hertfordshire, UK) Anti-Bcl-2 (Cell Signalling, Hertfordshire, UK) Anti-HO-1 (Cell Signalling, Hertfordshire, UK) Anti-NQO1 (Cell Signalling, Hertfordshire, UK) Anti-GAPDH (Abcam) Secondary horseradish peroxidase (HRP)-conjugated anti-rabbit (Sigma-Aldrich, Dorset, UK) Secondary horseradish peroxidase (HRP)-conjugated anti--mouse (Sigma--Aldrich, Dorset, UK)	1:500 1:500 1:1000 1:1000 1:1000 1:1000 1:1000 1:500 1:2000 1:2000

**Table 2:** Primers used for qPCR analysis.

Bax	Forward ^5′^TCAGGATGCGTCCACCAAGAAG^3′^ Reverse ^5′^TGTGTCCACGGCGGCAATCATC^3′^
Bcl-2	Forward ^5′^ATCGCCCTGTGGATGACTGAGT^3′^ Reverse ^5′^GCCAGGAGAAATCAAACAGAGGC^3′^
P-p44/42	Forward ^5′^ACACCAACCTCTCGTACATCGG^3′^ Reverse ^5′^TGGCAGTAGGTCTGGTGCTCAA^3′^
HO-1	Forward ^5′^CCAGGCAGAGAATGCTGAGTTC^3′^ Reverse ^5′^AAGACTGGGCTCTCCTTGTTGC^3′^
NQO1	Forward ^5′^CCTGCCATTCTGAAAGGCTGGT^3′^ Reverse ^5′^GTGGTGATGGAAAGCACTGCCT^3′^
p62	Forward ^5′^TGTGTAGCGTCTGCGAGGGAAA^3′^ Reverse ^5′^AGTGTCCGTGTTTCACCTTCCG^3′^
LC3-II	Forward ^5′^GAGAAGCAGCTTCCTGTTCTGG^3′^ Reverse ^5′^GTGTCCGTTCACCAACAGGAAG^3′^
Beta tubulin	Forward ^5′^CTGGACCGCATCTCTGTGTACT^3′^ Reverse ^5′^GCCAAAAGGACCTGAGCGAACA^3′^
GAPDH	Forward ^5′^TTGCCCTCAACGACCACTTT^3′^ Reverse ^5′^TGGTCCAGGGGTCTTACTCC^3′^
ACTB	Forward ^5′^CACCATTGGCAATGAGCGGTTC^3′^ Reverse ^5′^AGGTCTTTGCGGATGTCCACGT^3′^
RPL-5	Forward ^5′^ATGCTCGGAAACGCTTGGT^3′^ Reverse ^5′^GCGCAGACTATCATATCCCCC^3′^
